# Computed tomography pulmonary angiography in around-the-clock clinical care with individualised scan protocols: a 5-year observational study on incidence and causes of repeat scanning

**DOI:** 10.1007/s00330-025-12032-y

**Published:** 2025-10-18

**Authors:** Estelle C. Nijssen, Bibi Martens, Babs M. Hendriks, Hester A. Gietema, Joachim E. Wildberger, Cécile R. L. P. N. Jeukens

**Affiliations:** 1 Department of Radiology and Nuclear Medicine, Maastricht University Medical Center+, Maastricht, The Netherlands; 2 Cardiovascular Research Institute Maastricht (CARIM), Maastricht University, Maastricht, The Netherlands; 3 Research Institute for Oncology and Reproduction (GROW), Maastricht University, Maastricht, The Netherlands

**Keywords:** Computed tomography angiography, Pulmonary embolism, Multidetector computed tomography, Diagnostic imaging, Precision medicine

## Abstract

**Objectives:**

Elevated repeat-scanning rates are reported for CT pulmonary angiography (CTPA). Individualised protocols optimise contrast- and radiation-doses, but whether this affects repeat scanning is unknown. The current study evaluates repeat-CTPA in a 24/7, state-of-the-art clinical-care setting.

**Materials and methods:**

This is a retrospective observational single-centre study of consecutive CTPA acquired over a 5-year period during standard clinical care. The primary outcome is the repeat-scan rate. Repeat- and single-scan groups were compared for initial-scan characteristics (patient-related, CT-scanner, contrast-administration, kV-settings, regular hours/shifts, radiation-dose), and cumulative contrast- and radiation-doses. An expert radiologist panel retrospectively evaluated probable reasons for repeat scanning through visual, subjective assessment of initial-scan images.

**Results:**

CTPA repeat rate was 3.1% (139/4467). Repeat- and single-scan groups significantly differed: age (55 ± 18 vs. 63 ± 17 years; *p* < 0.001), Body Mass Index (27 kg/m^2^ (IQR 7) vs. 25 kg/m^2^ (IQR 6); *p* = 0.022), radiation-dose (141 mGy∙cm (IQR 73) vs. 121 mGy∙cm (IQR 70); *p* < 0.001). Cumulative contrast- and radiation-doses were: 96 mL (IQR 31) vs. 48 mL (IQR 22) (*p* < 0.001); 0.36 gI/kg (IQR 0.11) vs. 0.18 gI/kg (IQR 0.51) (*p* < 0.001); 272 mGy∙cm (IQR 69) vs. 121 mGy∙cm (IQR 70) (*p* < 0.001).

Retrospective expert-consensus reasons for repeat scanning were: 31/133 patient-related; 28/133 multifactorial; 12/133 contrast/scan-protocol; 4/133 operator-error; 2/133 unidentified. 56/133 (42%) initial scans were retrospectively deemed diagnostic-quality, and these significantly differed from other repeat-categories in patient characteristics age (51 ± 15 vs. 57 ± 19 years; *p* = 0.045) and sex (64.3% vs. 50.6% female; *p* = 0.045), and in contrast volume (48 mL (IQR 17) vs. 46 mL (IQR 24); *p* = 0.031).

**Conclusion:**

Individualised scan protocols yielded diagnostic images around the clock, with repeat scanning well within ranges published in the literature. Retrospective expert evaluation suggests repeat rates as low as 1.2% may be possible. Repeat- and single-scan groups significantly differed in patient characteristics, and repeat-scanning reasons were mostly patient-related. These results suggest further tailoring protocols to (younger, female) patients might be beneficial in helping to further reduce CTPA-repeats.

**Key Points:**

***Question*** *CT pulmonary angiography (CTPA) is subject to relatively high repeat-scanning rates, but it is not known how state-of-the-art CTPA and individualised protocols perform in clinical practice today*.

***Findings*** *During 5 years of clinical practice the repeat rate was 3%; retrospective expert image-evaluation suggests a repeat rate as low as 1.2% may be possible*.

***Clinical relevance*** *Repeat- and single-scan groups significantly differed in patient characteristics, and reasons for repeat scanning were mostly patient-related. Further tailoring protocols to (younger, female) patients may be the best focus to help reduce CTPA-repeats, improve safety, and reduce logistic burden*.

**Graphical Abstract:**

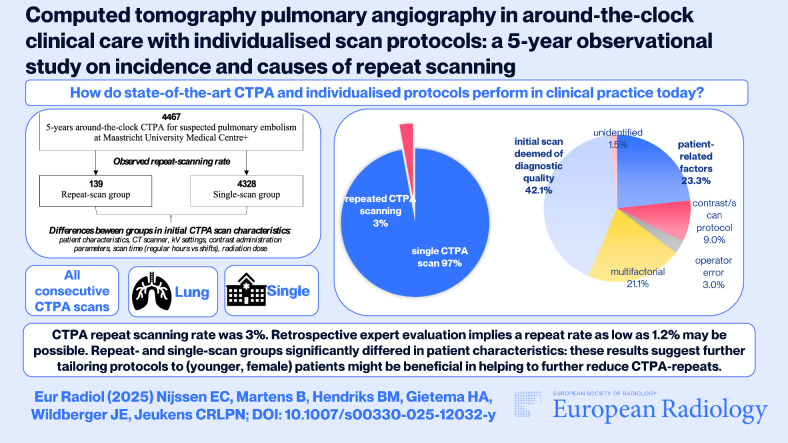

## Introduction

Due to high sensitivity and specificity, computed tomography pulmonary angiography (CTPA) is currently the diagnostic tool of choice for pulmonary embolism in the emergency setting [1–3]. However, CTPA comes with several challenges. The first challenge concerns exacting timing requirements for the acquisition of diagnostic-quality images. Intravenously administered contrast material (CM) must be at peak density in the pulmonary arteries during the scan, which usually lasts only seconds. Unsurprisingly, the reason for non-diagnostic CTPA scans most often reported in the literature is ‘poor contrast timing’ [4, 5]. Furthermore, although there is currently no widely accepted threshold in literature for adequate pulmonary arterial attenuation, peak attenuation in the pulmonary arteries must be sufficiently high to enable accurate embolism identification [6–8]. The second challenge of CTPA is overcoming patient-related factors such as motion-artefacts, cardiac parameters, inspiration or Valsalva breathing manoeuvres, which affect blood flow into the pulmonary arteries [9]. Enhancement of the pulmonary arteries may also differ between central and peripheral branches, especially in patients with parenchymal disease. All in all, CTPA is one of the most frequently rejected CT examinations: CTPA rejection rates may be as high as 11.2% where overall CT scan rejection rates are less than 2% [10]. When an initial CTPA scan is rejected, repeat scanning will usually be performed to attain diagnostic images. This not only imposes a logistical burden but will also increase patient CM and radiation doses.

Technical developments enabling faster scanning and long-established methods such as test bolus techniques have made acquisition of diagnostic CTPA scans increasingly straightforward [11–13]. On the other hand, continuous efforts are made to optimise scan- and CM administration protocols and to automate processes, with the aim to reduce individual radiation and CM doses whilst maintaining diagnostic image quality [14–23]. By dint of narrowing the optimum time window, such reductions increase the importance of correct timing and may increase repeat rates. Previous research at our centre demonstrated that individualised CM injection protocols, based on bodyweight and tube voltage, resulted in lower CM volumes and lower radiation doses whilst maintaining diagnostic attenuation of the pulmonary arteries in most patients [17–20, 23]. However, study settings differ from routine clinical practice, and the question arises how state-of-the-art CTPA, using these individualised protocols, performs with respect to repeat-scanning in clinical practice today.

The current study aim is to evaluate repeat CTPA scanning over a 5-year period during standard clinical care at an academic hospital, which provides local primary to (inter)national tertiary care and has a state-of-the-art radiology department using optimised and individualised scan and CM injection protocols.

## Materials and methods

### Study design and population

All patients received standard care. The local medical ethics committee waived the requirement for informed consent (METC 2020-1535-A-1).

This is a retrospective, observational, single-centre study of CTPA scans acquired around the clock during standard clinical care at Maastricht University Medical Centre+ (UMC+). All consecutive CTPA scans acquired for suspected pulmonary embolism between January 1, 2017 and January 1, 2022, were eligible for inclusion. This 5-year monitoring period of all consecutive CTPA scans was chosen to enable long-term observation of applying individualised protocols to CTPA in practice, avoiding bias through image-, operator- or patient- selection, and starting from the time when individualised protocols were fully implemented.

### CTPA protocols

Scan acquisition parameters and image reconstruction parameters were based on scanner automation and vendor recommendations. Contrast administration protocols were individually tailored based on patient bodyweight (CM volume and flow rate) and automated tube voltage (kV) selection as proposed by the scanner (CARE kV, Siemens) [17–23].

At Maastricht UMC+, three CT scanners were used for CTPA during the study period (Siemens Healthineers):

- Scanner 1. 2nd generation dual-source CT (DSCT; SOMATOM, Definition Flash)
- Scanner 2. 3rd generation DSCT (SOMATOM Force)
- Scanner 3. 64-slice multidetector-row CT (MDCT; AS 64)

Scan acquisition parameters were as follows: Automated tube current modulation (Care Dose 4D, Siemens) mAs_ref_ 120 for scanner 1, 105 for scanner 2, 131 for scanner 3; Automated tube voltage selection (Care kV, Siemens) kV_ref_ = 100 kV with kV-options 70, 80, 100, 120 and 140 kV, plus 90, 110, 130 and 150 kV for scanner 2; Pitch 1.8 for scanner 1, 1.3 for scanner 2, 1.4 for scanner 3; Collimation 128 × 0.6 for scanner 1, 192 × 0.6 for scanner 2, 64 × 0.6 for scanner 3.

On the SOMATOM Force, a single-source acquisition setup was chosen over the high-pitch dual-source mode [20]. This setup can be uniformly implemented across centres, including those without access to dual-source technology.

The radiation dose does not change between dual- and single-source modes when reference mAs remains constant, as is the case in our practice. Regarding scan speed, the SOMATOM Force compensates for the slightly lower pitch (1.3 compared to 1.8 on the Flash) through a faster rotation time (0.25 s vs. 0.285 s) and broader detector coverage. Consequently, both scanners achieve comparable overall volume coverage times, despite the different acquisition modes [17, 20].

Image reconstruction parameters were: Slice thickness 1 mm and increment 0.8 for scanners 1 & 3, 0.7 for scanner 2; Regular kernel (I26f for scanner 1, Bv40 for scanner 2, Br38 for scanner 3), Iterative reconstruction software SAFIRE (Sinogram Affirmed Iterative Reconstruction) for scanners 1 & 3, ADMIRE (Advanced Modelled Iterative Reconstruction) for scanner 2, and Strength level 3 - as advocated by the suppliers.

All patients received intravenous iodinated CM iopromide, with 300 or 370 mg iodine per mL (Ultravist, Bayer Healthcare). Individualised CM injection protocols were used: flow rates and CM volumes  were adjusted to specific patient bodyweight (continuous adaptation) and scanner-selected kV settings. To maintain stable iodine attenuation the 10-to-10 rule was applied, based upon previous research which showed that when tube voltage is reduced by 10 kV CM volume can be reduced by 10% and still yield diagnostic images (i.e., 10% reduction in iodine delivery rate per 10 kV decrease, and vice versa) [14, 20, 22, 23]. CM flow rate was calculated using kV-dependent iodine delivery rate (IDR) reduction percentages and bodyweight–adapted IDR values taken from the P3T software (Bayer), as has been validated in previous studies [14, 20, 22, 23]. For full details on the application of the 10-to-10 rule, see [20].

A test bolus was used to determine scan delay, with injection duration 2.5 s at the main-bolus flow rate. An 8-s injection duration was used for the main bolus, followed by 40 mL saline flush at main-bolus flow rate.

### Outcomes and data collection

The primary outcome was the incidence of repeated CTPA scans during standard clinical care. CTPA scans registered under the same unique accession number, or two or more CT-thorax scans within 8 days registered under the same unique patient identifier, were visually checked to verify repeat-scan status. Potential repeat CTPA scans were identified using CT acquisition metadata retrieved from a commercially available dose monitoring software (Radimetrics^TM^, Bayer).

Secondary outcomes include difference in characteristics between the initial CTPA scans of single-scan and repeat-scan groups: patient characteristics (age at time of scan, sex, bodyweight and -length, Body Mass Index (BMI)), CT scanner, kV settings, CM administration parameters (volume and flow rate), scan time (regular working hours during weekdays between 08:00–17:00, vs. other), and radiation dose (all retrieved using Radimetrics^TM^, Bayer). Only CM and radiation directly associated with imaging for pulmonary embolism (PE) evaluation were included.

CM parameters were monitored by a dedicated data acquisition programme (Certegra Informatics Solution; Bayer), and CM parameters and radiation dose, expressed as dose length product (DLP) in mGy∙cm, were retrieved using the abovementioned dose monitoring software. Total cumulative CM and radiation doses for pulmonary embolism evaluation received by repeat- and single-scan patients were also compared.

An expert panel retrospectively evaluated probable reasons for repeat scanning through visual, subjective assessment of initial-scan images. The expert panel included 3 radiologists with 28-, 19- and 7-years’ experience in thoracic imaging (J.W., H.G., and B.M.).

Panel members individually viewed images on a clinical picture archiving and communication system (PACS; Sectra Workstation, v23.1, Sectra AB). The most probable reason for repeat scanning was structured into six pre-defined categories: 1. Patient-related factors; 2. CM administration/scan protocol; 3. Operator error; 4. Multifactorial; 5. Unidentified (i.e., repeat was necessary, but the specific reason is unclear to the panel); 6. Initial-scan images were deemed of diagnostic quality (i.e., pulmonary embolism visible or ruled out with high confidence up to the subsegmental level). Expert-panel evaluation outcomes were based on consensus: the panel convened to arrive at a consensus category for each scan.

Subgroup analysis was done to compare initial-scan characteristics between category 6 (initial-scan diagnostic) and the other categories.

### Statistical analysis

Categorical data are presented as absolute numbers and percentages, continuous data as means with standard deviations (normal distribution), or medians with interquartile ranges (IQR). Differences in proportions between groups were tested using the Chi-square test. Continuous variables were compared using the t-test for independent samples (normal distribution) or the non-parametric Mann–Whitney *U*-test. Distributions of continuous variables were visually checked for normality by a histogram, and kurtosis and skewness were evaluated. In cases of missing data, available case analysis was done. *p*-values < 0.05 were considered to indicate statistical significance. Data was organised using Microsoft Excel (version 16.78, 2023; Microsoft Corporation); analyses were done using IBM SPSS Statistics for Macintosh (version 29.0; IBM Corporation).

## Results

**Fig. 1:**
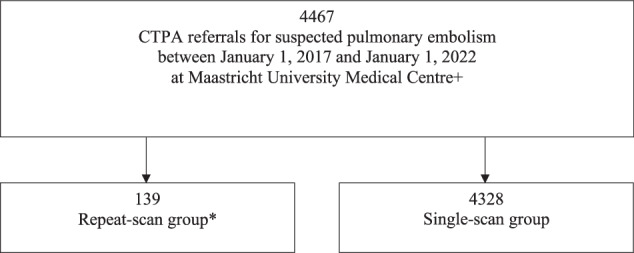
Study profile. CTPA, computed tomography pulmonary angiography. * A repeat scan was defined as two or more CTPA scans registered under the same unique accession number or two or more CT-thorax scans registered under the same unique patient within 8 days

Between the 1st of January 2017 and the 1st of January 2022, 4467 CTPA scans were acquired for suspected pulmonary embolism at Maastricht UMC+ (Fig. 1): 139/4467 (3.1%) were repeated.

### Characteristics of initial scans in repeat- and single-scan groups

Characteristics of initial CTPA scans in repeat- and single-scan groups are given in Table 1.

**Table 1:** Characteristics of initial scans in repeat- and single-scan groups

	Repeat scan *n* = 139	Single scan *n* = 4328	*p*-value
Patient characteristics^a^			
Age (years)	55 (±18)	63 (±17)	**< 0.001**
Sex (female)	78 (56.1%)	2358 (54.5%)	0.973
Bodyweight (kg)	80 (22)	75 (21)	0.062
	Range 42–120	Range 29–175	
Length (cm)	172 (±9)	170 (±10)	0.261
BMI (kg/m^2^)	27 (7)	25 (6)	**0.022**
Scanner			
1. Siemens Flash^b^	118 (84.9%)	3.827 (88.4%)	0.439
2. Siemens Force	15 (10.8%)	364 (8.4%)	
3. Siemens AS 64	6 (4.3%)	137 (3.2%)	
kV Setting			
70 kV	3 (2.2%)	138 (3.2%)	0.053
80 kV	40 (28.8%)	1519 (35.1%)	
90 kV	5 (3.6%)	75 (1.7%)	
100 kV	69 (49.6%)	2196 (50.7%)	
110 kV	1 (0.7%)	14 (0.3%)	
120 kV	21 (15.1%)	386 (8.9%)	
Time of scan			
Weekdays 08:00–17:00	82 (59.0%)	2412 (55.7%)	0.446
Contrast material^c^			
Volume administered (mL)	47 (21)	48 (22)	0.741
Iodine dose (gI/kg bodyweight)	0.17 (0.61)	0.18 (0.51)	0.777
Flow rate (mL/s)	4.6 (±1.0)	4.7 (±1.1)	0.643
Radiation dose			
DLP (mGy∙cm)	141 (73)	121 (70)	**< 0.001**

Data are in *n* (%), mean (±SD) or median (IQR). Data are from initial CTPA scans only. Bold type indicates statistical significance

^a^ Bodyweight was available for *n* = 90/139 and *n* = 2562/4328 patients; body length for 111/139 and 2451/4328 patients; BMI for 82/19 and 2449/4328 patients

^b^ This scanner is located closest to the emergency department

^c^ Data on contrast volume was available for *n* = 120/139 and 3551/4328 patients, data on CM iodine dose and flow rate were available for 80/139 and 2422/4328 patients

Repeat- and single-scan groups significantly differed in patient age (55 ± 18 vs. 63 ± 17 years; *p* < 0.001), patient BMI (27 kg/m^2^ (IQR 7) vs. 25 kg/m^2^ (IQR 6); *p* = 0.022), and radiation dose (141 mGy∙cm (IQR 73) vs. 121 mGy∙cm (IQR 70); *p* < 0.001). Although median bodyweight was higher in the repeat-scan group, distributions did not significantly differ (80 kg (IQR 22, range 42–120 kg) vs. 75 kg (IQR 21, range 29–175 kg); *p* = 0.062).

Scanner use was similar across groups (*p* = 0.439), and repeat rates were similar between scanners (scanner 1: 118/3945, 3.0%; scanner 2: 15/379, 4.0%; scanner 3: 6/143, 4.2%; *p* = 0.348). Although the highest setting of 120 kV was used more often in the repeat-scan group (21/139, 15.1% vs. 386/4328, 8.9%), the distribution of kV settings did not significantly differ between groups (*p* = 0.053). Most scans were done at 100 kV (repeat-scan group 69/139, 49.6%; single-scan group 2196/4328, 50.7%), followed by 80 kV (repeat-scan group: 40/139, 28.8%; single-scan group: 1519/4328, 35.1%).

A little over half the scans in both groups were done during regular working hours (repeat-scan group: 82/139, 59.0%; single-scan group: 2412/4328, 55.7%; *p* = 0.446). Initial-scan CM volume and flow rate were similar across groups: 47 (IQR 21) vs. 48 (IQR 22) mL (*p* = 0.741); 4.6 ± 1.0 vs. 4.7 ± 1.1 mL/s (*p* = 0.643). Initial-scan DLP was significantly higher in the repeat-scan group: 141 mGy∙cm (IQR 73) vs. 121 mGy∙cm (IQR 70; *p* < 0.001).

### Total cumulative CM and radiation doses

Both cumulative CM and radiation doses were significantly higher in the repeat-scan group than in the single-scan group (Table 2): CM volume 96 mL (IQR 31) vs. 48 mL (IQR 22; *p* < 0.001); iodine dose 0.36 gI/kg (IQR 0.11) vs. 0.18 gI/kg (IQR 0.51), *p* < 0.001; DLP 272 mGy∙cm (IQR 69) vs. 121 mGy∙cm (IQR 70; *p* < 0.001).

**Table 2:** Cumulative contrast and radiation received in repeat- and single-scan groups

	Repeat-scan group *n* = 139	Single-scan group *n* = 4328	*p*-value
Contrast^a^			
Total volume (mL)	96 (31)	48 (22)	**< 0.001**
Total iodine dose (gI/kg bodyweight)	0.36 (0.11)	0.18 (0.51)	**< 0.001**
Radiation			
DLP (mGy∙cm)	272 (69)	121 (70)	**< 0.001**

Data are in median (IQR). Data are from all CTPA scans. Bold type indicates statistical significance

^a^ Data on contrast volume was available for 120/139 and 3556/4328 patients; data on iodine dose were available for 80/139 and 2424/4328 patients

### Reasons for repeat scanning

**Fig. 2:**
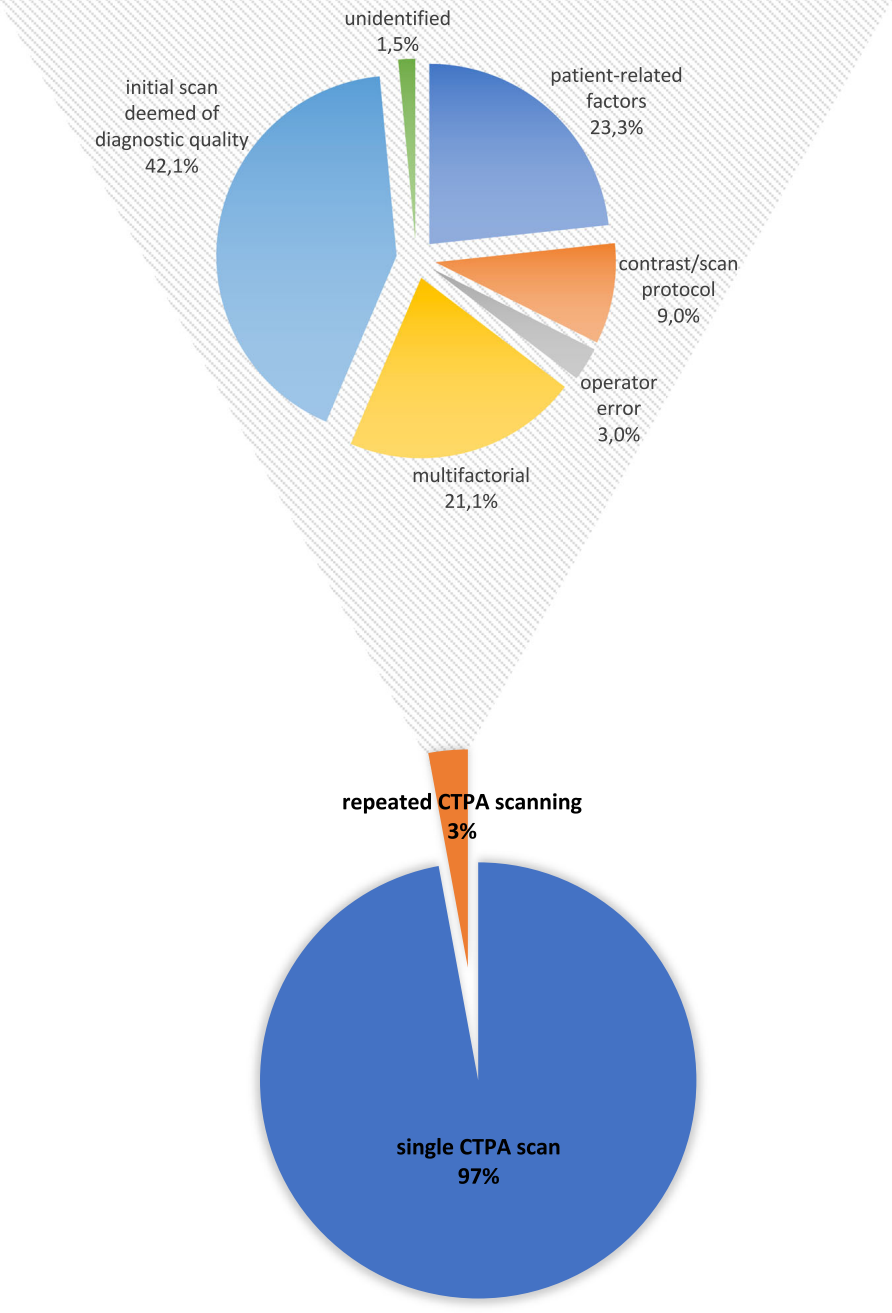
Retrospective expert consensus on reasons for repeat scanning. The expert panel consisting of three senior radiologists, retrospectively evaluated 133 cases from the repeat-scan group through visual, subjective assessment of initial-scan images, and categorised repeats into 6 pre-defined categories. Patient-related factors were Valsalva manoeuvres (including two pregnant women in their third trimester and one post-operative patient), inspiration artefacts, chronic thrombo-embolic pulmonary hypertension, severe emphysema, inability to move the arms out of the field of view (for example, after recent breast surgery), and six patients had significant stenosis of the venous inflow vessel (subclavian vein or superior vena cava) with extensive contrast filling of collaterals in the neck and the chest wall. Operator errors included incorrect patient weight and CM flow rate, not adjusting CM to changed kV settings, and incorrect timing

**Fig. 3:**
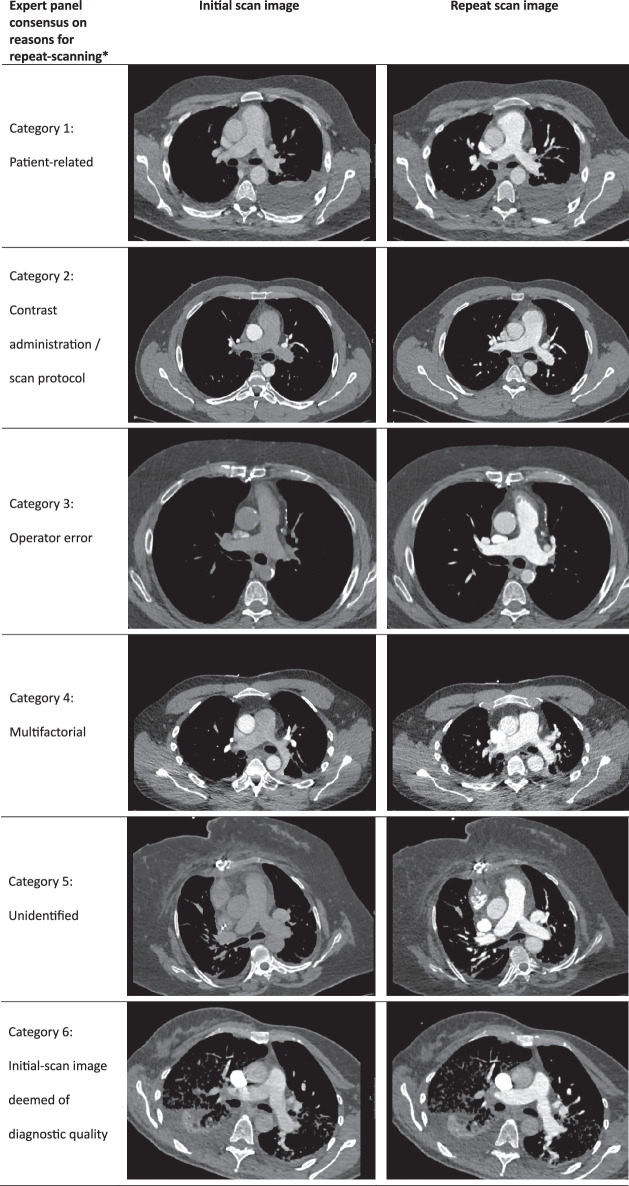
Examples of initial and repeated CTPA scan images. * An expert panel retrospectively evaluated probable reasons for repeat scanning through visual, subjective assessment of initial-scan images. The most probable reason for repeat scanning was structured into six pre-defined categories: 1. Patient-related factors; 2. CM administration/scan protocol; 3. Operator error; 4. Multifactorial; 5. Unidentified (i.e., repeat was necessary, but the specific reason is unclear to the panel); 6. Initial-scan images were deemed of diagnostic quality (i.e., pulmonary embolism visible or ruled out with high confidence up to the subsegmental level). Expert-panel evaluation outcomes were based on consensus

The expert panel retrospectively evaluated 133 cases from the repeat-scan group through visual, subjective assessment of initial-scan images (Fig. 2; examples of initial and repeated CTPA scan images are shown in Fig. 3). Retrospective expert-consensuses on reasons for repeat scanning were: 31/133 (23.3%) patient-related; 28/133 (21.1%) multifactorial; 12/133 (9.0%) CM/scan protocol; 4/133 (3.0%) operator error; 2/133 (1.5%) unidentified; and 56/133 (42.1%) initial scans were retrospectively deemed of diagnostic quality by the expert panel.

Initial scans that were retrospectively deemed diagnostic by the expert panel differed with statistical significance to the other repeat-categories (Table 3): patient age (51 ± 15 years vs. 57 ± 19 years, *p* = 0.045), sex (36/56, 64.3% vs. 42/83, 50.6% female, *p* = 0.045), CM volumes (48 mL IQR 17 vs. 46 mL IQR 24, *p* = 0.031). Although the highest setting of 120 kV was used less often in scans retrospectively deemed diagnostic (5/56, 8.9% vs. 16/83, 19.3%), the overall distribution of kV settings did not significantly differ between subgroups (*p* = 0.132).

**Table 3:** Characteristics of initial scans deemed diagnostic by the expert panel and other repeat scans

	Initial scan diagnostic *n* = 56	Other repeats *n* = 83	*p*-value
Patient characteristics^a^			
Age (years)	51 (±15)	57 (±19)	**0.045**
Sex (female)	36 (64.3%)	42 (50.6%)	**0.045**
Bodyweight (kg)	83 (22)	80 (9)	0.897
	Range 50–120	Range 42–110	
Length (cm)	171 ( ± 8)	173 (±10)	0.277
BMI (kg/m^2^)	28 (8)	27 (7)	0.370
Scanner			
1. Siemens Flash^b^	48 (85.7%)	64 (83.1%)	0.656
2. Siemens Force	6 (10.7%)	9 (11.7%)	
3. Siemens AS 64	2 (3.6%)	4 (5.2%)	
kV Setting			
70 kV	0 (0.0%)	3 (2.2%)	0.132
80 kV	15 (26.8%)	25 (30.1%)	
90 kV	2 (3.6%)	3 (3.6%)	
100 kV	33 (58.9%)	36 (43.4%)	
110 kV	1 (1.8%)	0 (0.0%)	
120 kV	5 (8.9%)	16 (19.3%)	
Time of scan			
Weekdays 08:00–17:00	28 (50.0%)	54 (65.1%)	0.215
Contrast material^c^			
Volume administered (mL)	48 (17)	46 (24)	**0.031**
Iodine dose (gI/kg bodyweight)	0.18 (0.48)	0.17 (0.74)	0.223
Flow rate (mL/s)	4.7 (1.1)	4.5 (±1.0)	0.304
Radiation dose			
DLP (mGy∙cm)	133 (64)	147 (96)	0.510

Data are in *n* (%), mean (±SD) or median (IQR). Data are from the initial CTPA scans only. Bold type indicates statistical significance

^a^ Bodyweight was available for *n* = 48/83 and *n* = 40/56 patients; body length for 61/83 and 48/56 patients; BMI for 44/83 and 36/56 patients

^b^ This scanner is located closest to the emergency department

^c^ Data on contrast volume was available for *n* = 48/56 and 67/83 patients, data on CM iodine dose and flow rate were available for 34/56 and 45/83 patients

## Discussion

This 5-year study showed a 3% CTPA repeat rate during standard clinical care, with retrospective expert-consensus putting reasons for repeat scanning mostly in the categories patient-related factors (23.3%) or multifactorial (21.1%). Unsurprisingly, cumulative CM and radiation doses were more than doubled for the repeat-scan group. Retrospective expert consensus would have it that 56 initial scans in the repeat-scan group (42%) were of sufficient diagnostic quality, suggesting a repeat rate as low as 1.2% may be possible, and that opportunities for further repeat-rate reduction may lie in identifying causes of these repeats.

Tailored scan and CM protocols were used in the current study, with flow rates and CM volumes adjusted to patient bodyweight and scanner-selected kV settings. These protocols have been shown to result in good image quality with low CM and radiation doses, but have not previously been evaluated in a routine round-the-clock clinical setting [20, 24–26]. The CTPA repeat rate of the current study falls within the 1.6%–6.3% range reported in the multi-centre study by Rose et al [10]. In that study, however, instead of continuous adaptation to bodyweight and kV settings, three pre-defined protocols were used based on bodyweight categories (< 140, 140–160, and > 160 kg), with fixed kV and CM volumes (140 kV and either 100 mL or 150 mL CM): repeat rates were highest for the > 160 kg bodyweight protocol (3% to 11%, *n* = 2315), followed by the 140–160 kg protocol (0.5% to 3.6%, *n* = 4497), and lowest for the < 140 kg protocol (0.8%, *n* = 373). In the current study, patient body weights mainly fall within the < 140 kg category (7 patients weighed over 140 kg and 2 over 160 kg), and current results are therefore probably best compared to those of the < 140 kg protocol used by Rose et al. In that light, the current repeat rate may be considered relatively high (3% vs. 0.8%). However, Rose et al’s data in the < 140 kg category was obtained from a single small sample (*n* = 373) and results may not be representative. On the other hand, if repeat scanning of initial scans that were retrospectively deemed diagnostic by the expert panel can in truth be avoided, the tailored approach of the current study would yield a repeat rate of 1.2%, which more closely resembles the 0.8% repeat rate found by Rose et al. More importantly, however, individualised protocols used in the current study led to much lower kV (over 90% of scans were done at ≤ 100 kV), and CM volumes (median volume 46 mL per scan) than the weight-category approach by Rose et al. Even the median *cumulative* CM dose in the repeat-scan group of this study was lower than the lowest dose in the study by Rose et al. This suggests that not only do tailored protocols result in impressive reductions in radiation and CM doses, but they can also yield as many diagnostic-quality images.

The main causes for repeating CTPA scans as determined by the expert panel were patient-related factors such as body habitus, motion artifacts, and Valsalva and/or cardiac output, which is in line with previous research [8, 9, 27, 28]. Of these, Valsalva manoeuvres may be avoided in this setting by omitting breathing instructions, since modern faster scanning has been shown to enable diagnostic acquisition during normal breathing [29].

Patients with repeated CTPA scans were 8 years younger on average (55 vs. 63 years, *p* < 0.001). The younger age in the repeat-scan group may in part be explained by larger pulmonary perfused blood volume in younger patients, probably due to higher lung densities [30]. Younger age of repeat patients may be relevant to the lifetime attributable cancer risk associated with radiation, especially for pregnant women [31]. BMI also significantly differed, with higher BMI in the repeat-scan group (27 kg/m^2^ (IQR 7) vs. 25 kg/m^2^ (IQR 6)). This result is in line with higher repeat rates in larger patients found in earlier studies [10]. The maximum 120 kV setting was used more often in the repeat-scan group (15.1% vs. 8.9%), but differences in kV-settings distributions did not reach statistical significance (*p* = 0.053).

Retrospective expert consensus on reasons for repeat scanning was that 42% of initial scans in the repeat-scan group were of sufficient diagnostic quality to diagnose or exclude pulmonary embolism. Interestingly, in these scans patients were significantly younger, more often female, and received higher CM volumes compared to other repeats, and the highest 120 kV setting was used less often (8.9% vs. 19.3%).

Future studies could elucidate whether, as the above differences suggest, further tailoring of protocols to (younger, female) patients may be beneficial, and whether that will help to avoid repeats.

Previous research, including an expert-panel evaluation of repeated CTPA scans, also reports diagnostic initial scans, albeit with a lower incidence (17% of CTPA scans reclassified from ‘suboptimal’ to ‘optimal’) [32]. Such panel evaluations are based on retrospective consensus between multiple experts, however, which is not the same as decision-making in clinical practice. Therefore, it is not expected that all such repeats can be avoided in day-to-day practice. Nevertheless, the current study shows that characteristics of initial scans for which the retrospective expert consensus was ‘diagnostic’ significantly differ from those of other repeated CTPA. Therefore, these are probably the first scans to address reducing repeat scanning.

All in all, current results support using individualised protocols: repeat rates during busy level-1 trauma centre clinical routine are well within expected ranges and remain stable in around-the-clock clinical practice, whilst contrast and radiation doses are significantly lower than those in other studies.

In this single-centre observational study, CTPA scans were acquired on three clinical systems from one specific vendor in an academic medical hospital with around-the-clock availability of specialised CT-technologists. Repeat rates at centres with lower availability of dedicated staff or using other systems may differ. On the SOMATOM Force, a single-source acquisition setup was used rather than the available high-pitch dual-source mode. Single-source acquisition is more widely available and can therefore be implemented uniformly across different centres, including those without access to dual-source technology, making the current study results more generalisable.

The current study is retrospective, and the true reasons for repeat scanning, as determined during clinical practice, are unknown: repeat-scanning reasons retrospectively determined by the expert panel may deviate from the bases of decisions made during clinical practice. Furthermore, because the current study's aim was to evaluate repeat scanning in daily clinical practice, clinical criteria for CTPA referrals, objective image quality (such as attenuation in regions of interest (ROI)), and technical aspects such as iterative reconstruction were not subject to investigation.

Although 4467 CTPA is a substantial dataset, we cannot exclude the possibility that a larger sample size will modify results to some degree. However, an observed repeat-scanning rate at a large medical centre providing local primary to (inter)national tertiary care over the duration of 5 years is expected to be representative.

Current data cannot be used to determine the percentage of CTPA images of sufficient diagnostic quality, all the more so because CTPA scans that were not repeated were not evaluated by an expert panel. However, the aim of this observational study was not to evaluate CTPA image quality but to determine how often repeat scanning was deemed necessary during routine clinical practice. If CTPA were not repeated in clinical practice, we may surmise that either the initial scan was fully diagnostic, or the initial scan was of lesser quality but sufficient for the clinical indication (e.g., excluding large pulmonary emboli).

Data on treatment strategies and patient outcomes, and therefore the clinical impact of repeat scanning, were beyond the scope of the current study. This is an important aspect of repeat-scanning that should be addressed in future research. Furthermore, whereas pregnancy may (in part) explain the higher percentage of young women seen in repeats, pertinent data were not available in this study. Future studies with prospective designs could provide greater insight into these topics.

Expert panel image quality evaluation remains subjective and cannot reflect day-to-day clinical decision-making. In addition, because image quality is usually assessed visually during clinical workflow, rather than through quantitative metrics, visual evaluation was the method chosen for expert panel review. Future studies may consider including objective image quality parameters such as ROI-based attenuation, but these may offer limited added value in this context: there is currently no widely accepted threshold in literature for adequate pulmonary arterial attenuation, and inadequate attenuation does not always mean diagnostically insufficient. Even in cases with relatively low intravascular contrast enhancement, a scan can still be considered diagnostically adequate. For example, images may be of sufficient quality when using alternate windowing, or pulmonary embolism is clearly visible and can be confidently diagnosed despite lower contrast.

In the future, artificial intelligence (AI) might help to increase the number of diagnostic CTPA and reduce repeat scanning [33–37]. Interestingly, Cheikh et al stress the use of AI to ensure negative findings in poor image quality CTPA [38], and Hagen et al used AI on unenhanced CT for improved detection of small pulmonary embolism [39].

## Conclusion

Individualised scan protocols yielded diagnostic image acquisition around the clock, with a repeat rate well within the ranges published in the literature. Furthermore, expert evaluation suggests a repeat rate as low as 1.2% may be possible. Cumulative contrast- and radiation dose increases were substantial after repeat scanning. Repeated scans significantly differed from single scans in patient characteristics, and reasons for repeat scanning were mostly patient-related. Future studies aiming to reduce CTPA repeats even further, which in turn would further improve CTPA safety and logistic burden, might do well to evaluate additional tailoring of protocols to (younger, female) patients.

## Abbreviations

BMI: Body Mass Index
CM: Contrast material
CT: Computed tomography
CTPA: Computed tomography pulmonary angiography
UMC: University Medical Centre
